# A retrospective study of 96 cases comparing x-ray radiography and MRI for diagnosing paediatric subacute osteomyelitis

**DOI:** 10.3389/fradi.2026.1744940

**Published:** 2026-04-01

**Authors:** Elio Paris, Ahmer A. Khan, Giacomo De Marco, Anne Tabard-Fougère, Oscar Vazquez, Christina Steiger, Romain Dayer, Dimitri Ceroni

**Affiliations:** 1 Faculty of Medicine, University of Geneva, Geneva, Switzerland; 2 Pediatric Orthopedics and Traumatology Unit, Geneva University Hospitals and University of Geneva, Geneva, Switzerland

**Keywords:** osteomyelitis, subacute, hematogenous, magnetic resonance imaging, conventional radiography, performance analysis, classification

## Abstract

**Background:**

Subacute hematogenous osteomyelitis (SAHOM) presents a diagnostic challenge, requiring robust validation of imaging accuracy.

**Purpose:**

To determine the superior diagnostic performance of MRI vs. radiography (x-ray) in detecting and classifying SAHOM.

**Methods:**

This retrospective study included 96 proven SAHOM cases (2000–2025). Demographic data, involved bones, and microbiological results were collected. Two independent readers assessed x-Ray and MRI for detection of SAHOM, and classified lesions using the modified Roberts classification. Inter-reader disagreements were resolved by consensus. Sensitivity of x-Ray was evaluated against MRI as the reference standard.

**Results:**

x-ray radiographs and MRI from 96 proven cases of SAHOM involving 49 males and 47 females (mean age 47.1 ± 47.6 months) were evaluated. MRI was markedly more sensitive, with significantly more correct imaging findings than radiography for detecting the features of SAHOM (100% vs. 47.9%). Moreover, 21.3% of the SAHOM lesions on x-ray radiography were misclassified. Radiography's limitations were most pronounced for lesions of the spine, tarsal/carpal bones, pelvis, and epiphysis, as well as for infections caused by *Kingella kingae* (*K. kingae*).

**Conclusions:**

MRI is a more effective method than x-ray radiography for diagnosing SAHOM; it reveals lesions with higher definition and enables their more precise classification. This is especially true of lesions involving the spine, pelvis, tarsal or carpal bones, and the epiphysis, or when SAHOM is caused by *K. kingae*. MRI also provides much better imaging of the involvement of growth cartilage and damage to articular cartilage.

## 1 Introduction

Subacute haematogenous osteomyelitis (SAHOM) is an infectious process characterised by moderate localised bone pain, mild or no systemic clinical manifestations, few confirmatory laboratory results and positive radiological findings (1–14). According to King and Mayo, any osseous infectious process lasting more than 2 weeks, but less than 3 months, without acute symptomatology, can be referred to as SAHOM (1). Currently, cases of SAHOM are typically linked to a particular host–pathogen relationship and manifest differently due to the lower virulence of the causative organism, greater host resistance or prior antibiotic exposure (3, 6, 7, 12–14). This form of haematogenous osteomyelitis, which mainly affects children, must be distinguished from other forms, such as chronic recurrent multifocal osteomyelitis and the SAPHO syndrome (synovitis, acne, pustulosis, hyperostosis and osteitis) (14, 15).

Because of its mild symptoms, the inconsistency of supportive laboratory data and the initially subtle radiological changes, SAHOM is a little-known nosological entity, or sometimes not known at all. For this reason, accurate diagnosis may be significantly delayed, allowing lytic bone lesions to develop, progress and become clearly visible on radiographs (x-Ray).

In the late 1990s, magnetic resonance imaging (MRI) technologies gradually replaced bone scintigraphy for investigating paediatric osteoarticular infections (OAIs) (16, 17). During the 2000s, referral centers used MRI in their work-ups for cases of suspected osteomyelitis and spondylodiscitis, thus facilitating the surgical decision-making process (18–24). More recently, it has become evident that the quality of care for children with OAIs has been significantly improved by more efficient and accurate diagnosis and by faster, more effective treatments (16). MRI is now an established part of work-ups for paediatric OAIs, acting as a supplementary examination for determining their extent and location more precisely, especially for delayed, complicated or atypical cases (17, 25–27).

In T1-weighted images, lesions due to SAHOM have a lower signal intensity than healthy bone, whereas in T2-weighted images, signal intensity is higher, but usually with a lower-intensity rim due to sclerotic bone. MRI has thus led to the earlier detection and more precise localisation of SAHOM (28). Specific signs of the condition, like the penumbra sign, have even been described using MRI, supporting diagnoses of SAHOM and helping to exclude the presence of a tumor (29, 30). However, some authors remain reluctant to recommend performing this examination for suspected SAHOM, especially in young children with normal radiographs, no fever and only mild osteoarticular symptoms, but mainly because MRI usually requires their sedation.

Thus, this study aimed to determine the superior diagnostic performance of MRI over x-ray in detecting and correctly classifying SAHOM.

## 2 Materials and methods

### 2.1 Study design and patients

After approval by the cantonal institutional ethics committee, the medical charts of patients aged from 1 day to 16 years old admitted to our institution with SAHOM from January 2000 to June 2025 were retrospectively reviewed. Diagnosis codes for osteomyelitis, septic arthritis with concomitant osteomyelitis, and SAHOM were all reviewed and used to identify potentially relevant cases of interest from the institution's electronic medical records. Only confirmed cases of SAHOM were included. On this subject, we used the Jansson criteria to exclude chronic nonbacterial osteomyelitis (CNO) (31). This algorithm comprises four major and six minor criteria, and it is used to diagnose chronic nonbacterial osteomyelitis, requiring a total score for classification. Key features include sterile, chronic, multifocal bone lesions, often accompanied by skin manifestations such as pustulosis. Finally, we also used the specific criteria summarized in Table 1 to define more precisely SAHOM.

**Table 1:** Diagnostic criteria for subacute hematogenous osteomyelitis (SAHOM) used in this study.

Category	Criteria
Temporal characteristics	Duration of symptoms >2 weeks but <3 months
	Observation time <3 months
Lesion characteristics	Monofocal lesion
	Isolated osteolytic bone lesion
	No hyperostosis
Clinical presentation	Moderate and localized bone pain
	None of few systemic manifestations
Patient history	No family history: grade 1 or 2 relatives with CNO or autoimmune diseases
	Skin manifestations: No clinical evidence of palmoplantar pustulosis (PPP) or psoriasis
Laboratory & microbiology	Laboratory results: None or few contributory findings
	Positive microbiology: Blood cultures, bone sample cultures, or PCR tests are positive

### 2.2 Epidemiological investigations

Epidemiological data were collected based on age, sex and the bones involved. Clinical and paraclinical investigations included temperature at admission and laboratory values such as white blood cell (WBC) count, platelet count, erythrocyte sedimentation rate (ESR) and the serum C-reactive protein (CRP) level.

### 2.3 Microbiological work-up

Blood cultures and bone or joint aspirate samples had been sent to the laboratory for immediate inoculation before starting antibiotic therapy. After 2007, two polymerase chain reaction (PCR) assays—broad-range PCR and *K. kingae*-specific real-time PCR—started to be performed on bone aspirate samples to identify the bacteria involved when standard cultures were negative. In some cases, PCR assays were also performed on blood samples. Since September 2009, our institution has also performed oropharyngeal swab PCR assays for children aged 6–48 months old. This has been shown to be a simple technique for detecting *K. kingae* rtx toxin genes in the oropharynx, and it provides strong evidence that this microorganism is responsible for an OAI, or even stronger evidence that it is not (32).

### 2.4 Radiological investigation

Patients eligible for the present study had to have sustained an osseous infectious process lasting more than 2 weeks, with no acute symptomatology, few confirmatory laboratory results and a complete radiological file suggestive of SAHOM. As a rule, participants had to have undergone x-ray radiographic imaging at or before admission, and MRI had to have been performed before or within 48 h of hospitalization. In this clinical context and considering current investigative methods, MRI is the considered the gold standard for the investigation of osteomyelitis and was thus considered as the reference standard.

Images were acquired (i) at 1.5-T (Siemens Healthcare GmbH, Erlangen, Germany with successive versions Symphony, Avanto, and finally Sola) using three-dimensional Short Tau Inversion Recovery (STIR) with T1-weighted turbo spin-echo (one longitudinal plane), (ii) in two orthogonal planes using T2-weighting and fat suppression, STIR (longitudinal plane), and water-only fast spin-echo T2-weighted Dixon sequences (axial plane), and (iii) using diffusion weighted imaging (axial plane) and post-contrast injection T1-weighted spin echo with frequency-selective fat saturation (two orthogonal planes). Postcontrast sequences were obtained after the injection of 0.2 mL/kg of gadoteric acid (Dotarem). During the studied period, the imaging protocols remained relatively unchanged. Only the contribution to the injected sequences has been modified over the past few years. Currently, radiologists are making the final choices on the most appropriate sequences and contrast enhancement materials to use, adapting them to the clinical scenario. Of the MRIs performed, 71 required sedations with anaesthesiologic assistance.

The report of radiological examinations prepared by radiologists at the time of their execution was recorded insofar as the diagnosis of SAHOM was based primarily on these documents. In addition to these information's contained in the patients’ computerized records, radiographs and MRI were independently and retrospectively reviewed by two independent readers; a senior paediatric orthopaedist (D.C.), and a medical resident (E.P.). Lesions due to SAHOM were distinguished using the Roberts’ classification, as modified by Ross (6, 33), first by using the radiographs and then using the MRI. The agreement between the results of these two imaging methods for classifying lesions due to SAHOM was then accurately established. In case of a disagreement between the readers, the the images were analysed together to reach a consensus opinion.

### 2.5 Statistical analysis

We analysed the characteristics of our entire population of eligible patients with SAHOM together. The normality of the distributions of their clinical manifestations and laboratory test results was evaluated using the normal Q-Q plot and the Shapiro–Wilk test. The sensitivity with which x-ray radiographs detected lesions due to SAHOM was evaluated using MRI as the method of reference, as it is currently considered as the gold standard imaging technology for identifying all similar infectious lesions. Patients were excluded if any of the key medical data were missing. Interobserver agreement was assessed using Cohen's kappa (κ) coefficient with 95% confidence intervals (CI). The strength of agreement was interpreted according to Landis and Koch: <0.00 = poor, 0.00–0.20 = slight, 0.21–0.40 = fair, 0.41–0.60 = moderate, 0.61–0.80 = substantial, and 0.81–1.00 = almost perfect.

The primary comparison focused on the detection rates of SAHOM. The detection proportion with its corresponding 95% confidence interval was calculated using the Wilson score method using the *binom.test* function in R. A prospective power calculation indicated a minimum of 23 cases was required to detect a 30% difference in sensitivity (90% vs. 60%) with 80% power (*α* = 0.05); our cohort of 96 provided ample power.

Statistical analysis was performed using R v.4.2.2 software (R foundation for statistical computing) with the RStudio interface (RStudio Team 2016; RStudio). Statistical significance was set at *p* < 0.05. All analyses were performed by a dedicated biostatistician (A.T-F).

## 3 Results

### 3.1 Epidemiology and skeletal distribution

**Figure 1:**
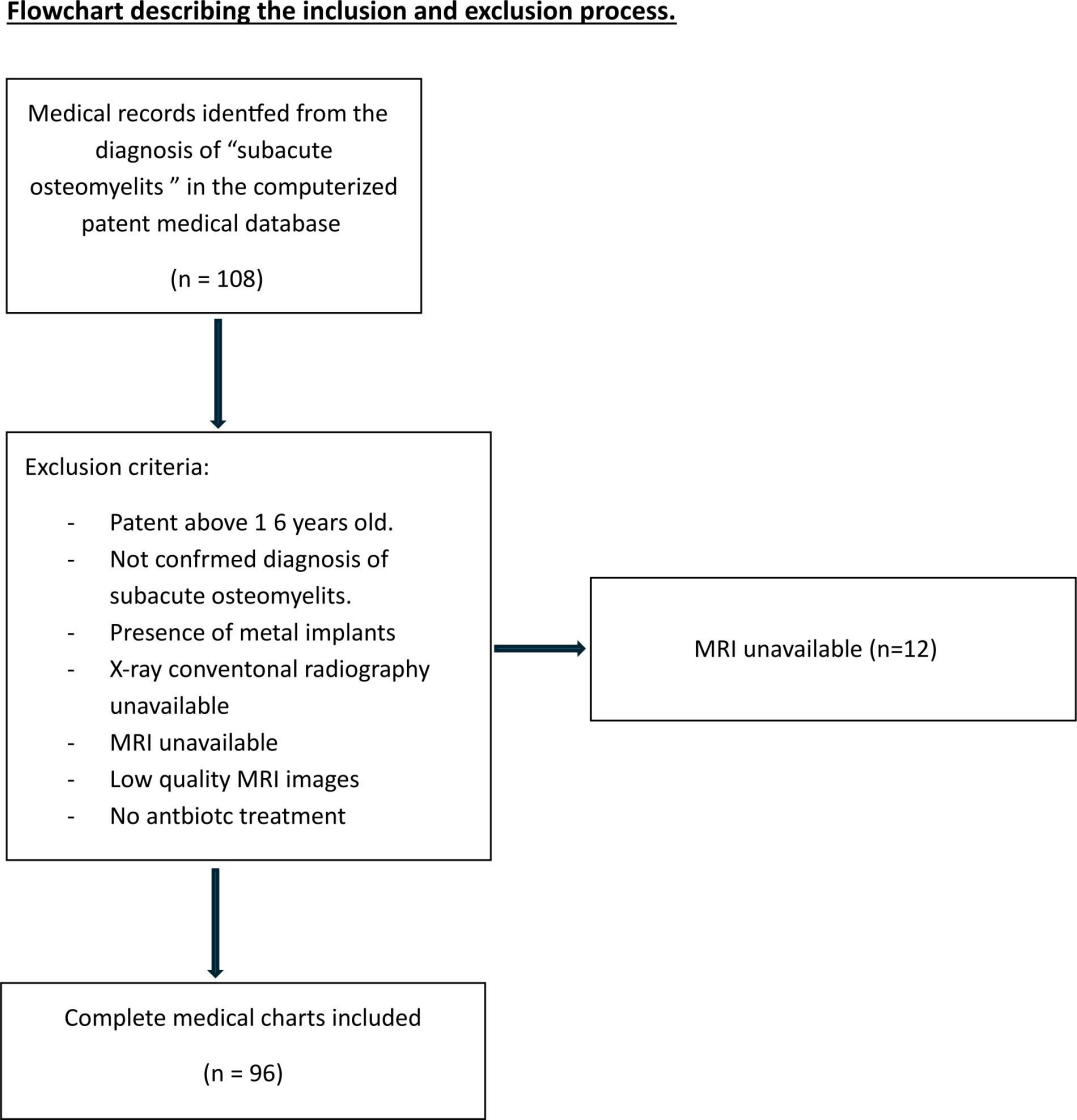
Flowchart of the included patients.

Of the 108 children treated for a confirmed SAHOM in our hospital, 96 had records with a complete radiological assessment and were eligible for inclusion in the study (12 cases without MRI) (Figure 1). The 47 girls and 49 boys had a mean age of 47.1 ± 47.6 months (Table 2). No cases were reported in children under 6 months, and 65 (67.7%) were younger than 48 months old at the onset of infection. SAHOM was most prevalent in the 13–24-month-old age group, with 34 children representing 35.4% of the sample. SAHOM mostly affected long bones (50 cases), tarsal or carpal bones (13 cases), flat bones (5 cases) and the patella (3 cases), with the remaining 26 cases affecting the spine. Lesion locations are summarized in Table 2.

**Table 2:** Population demographics and locations of the 96 included patients with confirmed subacute osteomyelitis.

Outcomes	Descriptive data
Demographics	
Age, months, mean (SD) [min; max]	47.1 (47.6) [6.0; 185.0]
Females, *n* (%)	47/96 (49%)
Locations	
Spine, *n* (%)	26 (26.8%)
Femur, *n* (%)	22 (22.7%)
Carpal & tarsal bones, *n* (%)	13 (13.4%)
Tibia, *n* (%)	9 (9.3%)
Fibula, *n* (%)	5 (5.2%)
Humerus, *n* (%)	5 (5.2%)
Ulna, *n* (%)	4 (4.1%)
Pelvis, *n* (%)	4 (4.1%)
Patella, *n* (%)	3 (3.1%)
Radius, *n* (%)	3 (3.1%)
Long bone in the feet, *n* (%)	2 (2.0%)
Rib, *n* (%)	1 (1.0%)

SD, standard deviation; min, minimum; max, maximum.

### 3.2 Clinical and inflammatory markers

When considering appropriate cut-off values (Table 3), WBC counts were normal (<12,000 cells/µL) in 77 cases (80.2%), with a mean of 9,520 ± 2,830 cells/µL (range: 1,600 to 19,000 cells/µL). CRP values were normal (<10 mg/L) in 55 cases (58.9%), with a mean value of 30.2 ± 17 mg/L in the other 39 cases. When measured (80 cases), the ESR was >20 mm/h in 26 cases (32.5%), with a mean value of 29.8 ± 17.6 mm/h (range: 4–95 mm/h).

**Table 3:** Distribution of clinical and laboratory parameters of the 96 included patients with confirmed subacute osteomyelitis.

Outcomes	*N* (missing)	Mean (SD)	Median	Min; Max
Temperature at admission, °C	96 (0)	37 (0.7)	37	35.8; 39.6
WBC count, 1,000 //µL	95 (1)	9.5 (2.8)	9.2	1.6; 19.0
Platelet count, 1,000 //µL	95 (1)	402.7 (112.7)	407	127; 755
CRP level (mg/L)	95 (1)	14.9 (21.3)	5.0	0.4; 95.0
ESR (mm/h)	80 (16)	29.8 (17.6)	26.0	4.0; 95.0

SD, standard deviation; IQR, interquartile range; Min, minimum; Max, maximum; CRP, C-reactive protein; WBC, white blood cell; ESR, erythrocyte sedimentation rate.

### 3.3 Bacteriological investigations

The usual blood or bone sample cultures detected a pathogen in 18 cases (18.8%) of children investigated bacteriologically. When combining the results of blood cultures, the usual bone sample cultures and the different PCR assays, a pathogen was detected in 46 cases overall (47.9%). Finally, 33 children's oropharyngeal swabs were positive for *K. kingae* rtx toxin genes. Of these, 15 children were highly suspected of having a *K. kingae*-induced SAHOM and could potentially be added to the list of patients for whom a pathogen was identified. The identification of a pathogen could thus be reasonably claimed in a total of 61 cases (63.5% of children). The list of pathogens responsible for cases of SAHOM is listed in Table 4.

**Table 4:** List of pathogens responsible of the subacute osteomyelitis (*n* = 96 patients).

Pathogen	Descriptive data
*Kingella kingae*, *n* (%)	29 (30.2%)
*Suspicion K. kingae*, *n* (%)	22 (22.9%)
MSSA, *n* (%)	9 (9.4%)
*Staphylococcus epidermis*, *n* (%)	3 (3.1%)
*Streptococcus pneumoniae*, *n* (%)	3 (3.1%)
*Streptococcus agalactiae B*, *n* (%)	1 (1.05%)
*Moraxella lacunata*, *n* (%)	1 (1.05%)
*Mycobacterium tuberculosis*, *n* (%)	1 (1.05%)
Gram (-), *n* (%)	1 (1.05%)
No germ, *n* (%)	26 (27.1%)

### 3.4 Radiological investigations

**Figure 2:**
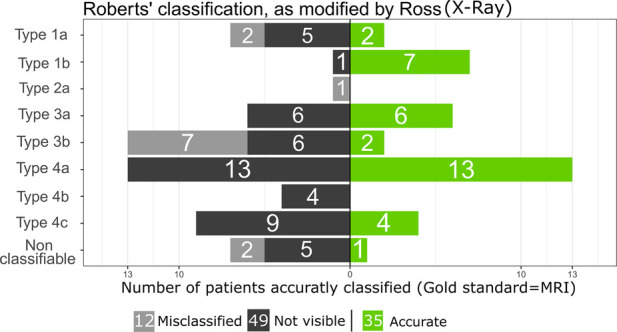
Distribution of the roberts’ classification, as modified by ross using x-ray, with the number of accurate patients (*n* = 35/96) in green (gold standard = MRI).

SAHOM could be diagnosed based on x-ray radiography in 47 cases, whereas every MRI was interpreted as positive. A disagreement in the recognition of the lesion between the 2 observers was noted in 3 cases (3/96; 3.1%) on conventional radiography [inter-observer reliability k = 0.943; IC 95% = (0.864, 1.000)], but detection rate of SAHOM on MRI were similar for the 2 observers [inter-observer reliability k = 100%; 95% = (96.4%, 100%)]. While MRI demonstrated a perfect detection rate (100%, 96/96), x-ray radiography identified only 47 of 96 (47.9%) cases (95% CI: 38.9%–59.2%). The non-overlapping confidence intervals indicated the statistically significant superior performance of MRI. The anatomical distribution and specifications of cases of SAHOM according to Roberts’ classification, as modified by Ross, are described in Figure 2. In 5 cases, there was disagreement regarding the classification of the lesion on conventional radiography [inter-observer reliability k = 0.924; IC 95% = (0.858, 0.989)] between both readers, while only one case resulted in misclassification with MRI [inter-observer reliability k = 0.986; IC 95% = (0.958, 1.000)]. After reaching a consensus on the interpretation of radiological investigations, the most frequently encountered lesions according to this classification were types 4a, 3a, 3b and 4c. x-ray radiograph analysis missed cases (not visible) of 4b, 3b, 4c, 4a and 3a-type lesions, namely those affecting the pelvis [4b: 4/4 (100%)], transphyseal lesions (3b: 6/15 (40%), carpal or tarsal bone lesions [4c: 9/13 (69.2%)], spinal lesions [4a: 13/26 (50%)] and epiphyseal lesions [3a: 6/12 (50%)]. When linked to a causative agent, nearly half (46.9%) of the cases with unrecognised SAHOM lesions were found to be due to *K. kingae*. In addition to unrecognised lesions, it appeared that 12 of the 49 lesions (24.5%) visible on x-ray radiographs were misinterpreted and frequently underestimated. In almost 60% (7/12) of the poorly classified cases, the lesions appeared to be transphyseal. Finally, even though all the lesions were visible on MRI, 8 of them (8.3%) could not be included in the modified Roberts classification.

**Figure 3:**
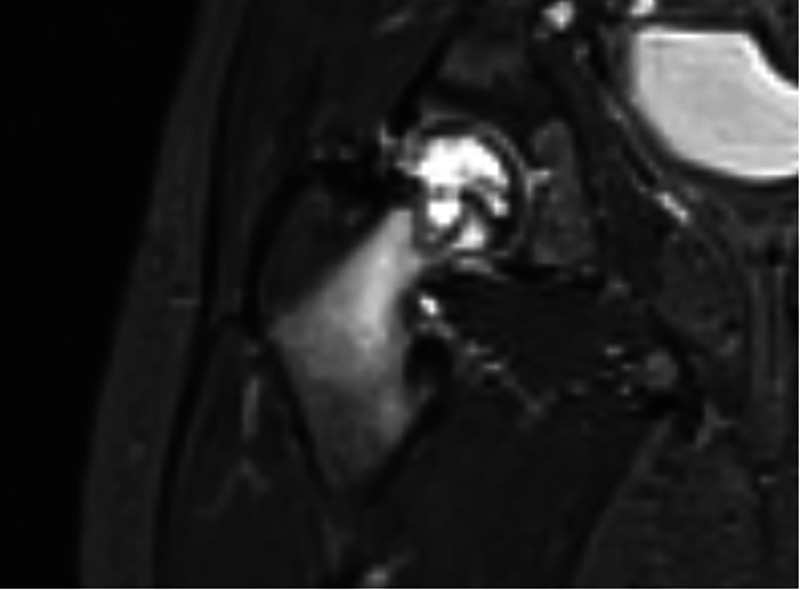
A 3-year-old child was referred to our emergency department with a limp for more than 3 weeks, without fever at admission, and the conventional radiography did not show any lesions. The laboratory tests were normal except for a sedimentation rate of 14 mm/h. MRI revealed a transphyseal lytic lesion of the femoral head without breach of the articular cartilage. A biopsy of the lesion under fluoroscopy detected the presence of *Kingella kingae* DNA.

**Figure 4:**
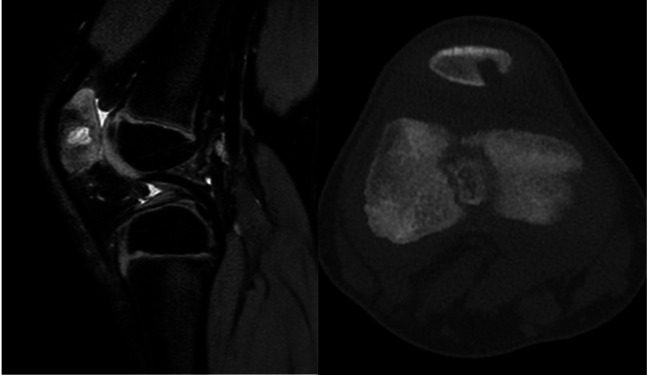
MRI revealed a lytic lesion of the patella in a 4-year-old girl, that was not visible on conventional radiographs. In this case, the condition of the articular cartilage was the key factor that determined the treatment strategy. The lytic lesion was due to *Moraxella lacunata*.

**Figure 5:**
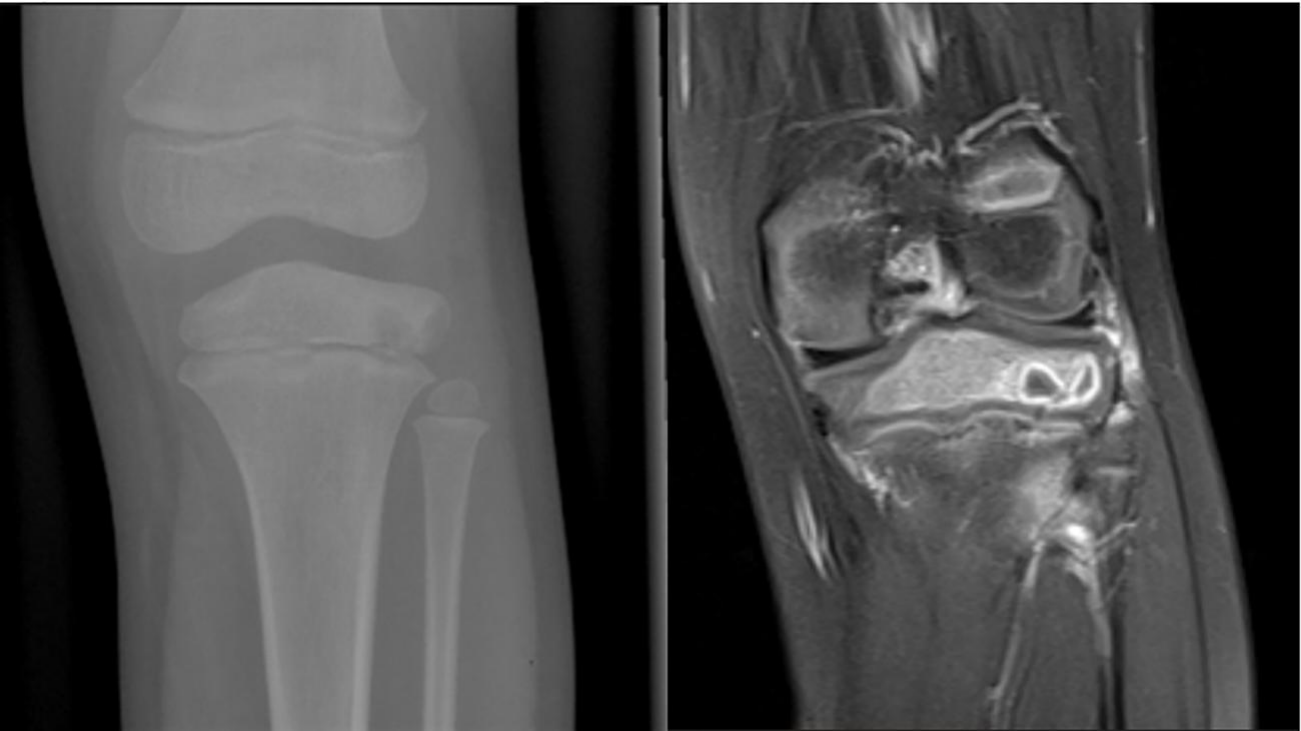
Conventional radiography and MRI of the knee in a 7-year-old child demonstrate a lytic lesion of the epiphysis due to *Staphylococcus aureus*. The MRI shows that the lesion was adjacent to the growth cartilage without crossing it. Note the presence of the “penumbra sign”; a rim of vascularized granulation tissue around the bone abscess cavity that has a higher T1 signal intensity than the cavity itself.

## 4 Discussion

Although each of the different imaging techniques involved in diagnosing SAHOM is important and complementary, the initial osseous changes may be so subtle that an accurate diagnosis may be delayed for a long time before a lytic bone lesion becomes obvious on x-ray radiographs. Indeed, a diagnosis of SAHOM is usually characterised by the presence of a Brodie abscess at the metaphysis. On x-ray radiographs, a Brodie abscess typically presents as an oval radiolucent lesion along the bone's longitudinal axis, with peripheral sclerosis within the metaphysis of long bones. This radiographical presentation usually follows a significant diagnostic delay, which is why MRI has established itself as the most efficient imaging method for paediatric OAI workups evaluating osteomyelitis, especially in delayed, complicated or atypical cases. Even though most authors now accept that MRI is superior to x-ray radiography for identifying SAHOM, no prior studies have been able to quantify this.

This study was, therefore, the first to attempt to compare the sensitivity of these imaging methods for diagnosing paediatric SAHOM. Firstly, our results showed that compared to MRI's 100% rate of SAHOM lesion detection, x-ray radiographs had a sensitivity of less than 50% (47.9%). Similarly, previous studies of chronic osteomyelitis in adults have highlighted that the radiological imaging of osteomyelitis varied according to the disease's progression through the acute, subacute, and chronic phases. x-ray radiography is not sensitive enough to assess the extent of osseous lesions within the first ten days of the onset of infection. The sensitivity of x-ray radiography during the disease's acute phase is estimated to be less than 5%, approximately 30% at one week, and up to 90% after 3–4 weeks (34). x-ray radiography is recognised as being able to reveal osteolysis as early as 10–21 days after the onset of the bone infection, but it may not be detectable until there is a loss of 30%–50% of the bone's mineral content (35, 36). Other authors, however, have suggested that lytic lesions only become radiographically apparent later, when 75% of the bone matrix has been destroyed (37). Our results showed that MRI infallibly recognised lesions due to SAHOM, confirming that it is the most sensitive (approaching 100%) radiological method for detecting osteomyelitis, even if its specificity is much lower (approximately 80%) (16, 38, 39). Indeed, a few studies have reported MRI's sensitivity in identifying bone lesions to be 81%–100%, with a specificity ranging from 67%–94% (16, 38, 40). Several reports have focused on specific MRI findings that may increase its specificity for recognising SAHOM. The ‘penumbra sign’, for example, has been described as a characteristic feature of MRI and is thought to be helpful in differentiating SAHOM from neoplasms (29).

Interestingly, our research suggested that some forms of SAHOM may be more difficult to detect on x-ray radiographs than others. According to the Roberts’ classification, as modified by Ross (33), most of the radiographs that initially missed cases of SAHOM were of the 4a, 4b, 4c, 3b and 3a types, representing infections affecting either the spine, the pelvis, the epiphysis, or the carpal or tarsal bones. In these cases, we suspect that the interpretation of x-ray radiographs can be hampered by over-projections, which make the detection of small lesions due to SAHOM particularly difficult. In addition to delays in detecting SAHOM, we observed that lesions were often poorly assessed on x-ray radiographs, leading to their misclassification. Indeed, our results showed that when osteomyelitis was detected by x-ray, more than 20% of cases were minimised and misclassified as a result. This applied especially to transphyseal lesions. Regarding MRI, SAHOM detection is excellent, but when compared to the modified Roberts classification, nearly 10% of the lesions fall outside the classification criteria. Finally, MRI provides a much better view of the involvement of growth cartilage and articular cartilage damage, which can condition and determine the orthopedic treatment.

The contribution of MRI is even more important as SAHOM is a distinct and difficult-to-diagnose form of osteomyelitis due to its insidious onset, mild symptoms, and inconsistent laboratory findings. Several previous studies had already made this observation, reporting abnormal ESRs in from 57.1%–81.3% of cases (8, 10–13), with abnormal WBC counts and CRP values in 0%–31.6% and 12.5%–39.8% of cases, respectively (8, 10–13). In addition, standard bacteriological analyses are not very effective at recognizing the pathogen responsible for SAHOM. In this regard, previous series had demonstrated that only 0%–12.5% of blood cultures had been able to detect the pathogen responsible for SAHOM (7–13), with 0%–75% of bone aspirate samples resulting in a bacteriological diagnosis (7–13). As a result, many subacute osteomyelitis cases are not initially reported, and it turns out consequently that many infections remain untreated, leading to the development of lytic lesions that may adversely affect the epiphysis or physis. Therefore, the use of MRI is crucial for early recognition and treatment, as it may help limit the formation of lytic lesions and prevent further damage.

The present study underlined that SAHOM preponderantly affects children aged from 6 to 48 months. In this series, 67.7% of cases of SAHOM occurred in patients in this age group, and *K. kingae* was over-represented as the causative bacterium. Analogously, a previous study by Spyropoulou et al. reported that approximately 85% of cases of SAHOM affected children younger than 4 years old, and that *K. kingae* was the only microorganism cultured in their series (13). In its infantile form, the clinical course of SAHOM can very easily be explained by *K. kingae*'s naturally low virulence. OAIs due to *K. kingae* are characterised by mild-to-moderate clinical and biological inflammatory responses, with few, if any, criteria evocative of SAHOM (41–46). Thus, for many children younger than 4, a diagnosis of osteomyelitis caused by *K. kingae* may be delayed, leading to potentially significant lytic lesions. However, in their early stages, lesions caused by *K. kingae* are more prone to being missed on x-ray radiographs.

Our study had some limitations. Its retrospective nature increased the risk of missing certain cases due to medical coding errors. This also increased the proportion of missing data and patients lost to follow-up. Some of the obvious lesions seen on radiographs were probably not subsequently investigated using MRI and were thus excluded from our study, affecting the sensitivity calculations for x-ray radiography. Furthermore, it is very likely that intra-reader variability may have been minimized by the design of the study—with only inclusion of confirmed diagnosis of PSAHO—since both readers knew even before reading the images that a lesion must be present and that they spent probably more time to try to find it on radiological investigations. Even if strict criteria aimed at excluding CNO were used in this study, it remains nonetheless that the difficulty that one may encounter in distinguishing SAHOM from CNO could also had affected the results of this study. Nevertheless, the descriptive material examined provided lots of information about the rates, characteristics and x-ray radiology investigations of SAHOM. These results should be confirmed and enriched by future real-world, multi-centre studies, which would enable an examination of larger numbers of patients and thus help to establish an algorithm for imaging/radiological investigations.

## 5 Conclusions

MRI is a more effective imaging method for finding and evaluating lesions due to SAHOM. x-ray radiography's sensitivity in the detection of lesions due to SAHOM was less than 50% (47.9%) compared to MRI. MRI provides much better definition and enables far more precise classification of lesions. This is especially true when the lesions affect the spine, pelvis and bone epiphysis, or when subacute osteomyelitis is caused by *K. kingae*. In addition, MRI provides a much better view of the involvement of growth cartilage and articular cartilage damage. However, future studies are needed to establish a new classification based on MRI to recognize all types of PSAHO.

## Data Availability

The raw data supporting the conclusions of this article will be made available by the authors, without undue reservation.
